# New target-HMGCR inhibitors for the treatment of primary sclerosing cholangitis: A drug Mendelian randomization study

**DOI:** 10.1515/med-2024-0994

**Published:** 2024-07-19

**Authors:** Jie Zhou, Yixin Xu, Haitao Wang, Zhilin Liu

**Affiliations:** Department of General Surgery, The Wujin Hospital Affiliated with Jiangsu University, Changzhou, Jiangsu Province, 213003, China; Department of General Surgery, The Wujin Clinical College of Xuzhou Medical University, Changzhou, 213003, China; Department of General Surgery, The Third Affiliated Hospital of Soochow University, Changzhou, 213003, China; Department of General Surgery, The Wujin Hospital Affiliated with Jiangsu University, No. 2, Yongning North Road, Changzhou, Jiangsu Province, 213003, China

**Keywords:** primary sclerosing cholangitis, statin, single-nucleotide polymorphism, mendelian randomization, efficacy

## Abstract

**Background:**

No intervention definitively extends transplant-free survival in primary sclerosing cholangitis (PSC). Statins, inhibitors of 3-hydroxy-3-methylglutaryl coenzyme A reductase (HMGCR), may enhance PSC prognosis, but their efficacy is debated.

**Methods:**

We analyzed HMGCR single-nucleotide polymorphisms from published genome-wide association studies using Mendelian randomization to assess the causal relationship between HMGCR and PSC risk. Effects of HMGCR were compared with proprotein convertase subtilisin kexin 9 (PCSK9) inhibitors, common lipid-lowering drugs, using coronary heart disease risk as a positive control. The inverse-variance weighted (IVW) method was the primary analysis, complemented by the weighted median method. Heterogeneity analysis, examination of horizontal pleiotropy, and leave-one-out sensitivity analysis were conducted for result robustness.

**Results:**

Genetically predicted HMGCR exhibited a pronounced detrimental effect on PSC in both the IVW method (odds ratio [OR] [95%] = 2.43 [1.23–4.78], *P* = 0.010) and the weighted median method (OR [95%] = 2.36 [1.02–5.45], *P* = 0.044). However, PCSK9 did not reach statistical significance. Moreover, all analyses passed through heterogeneity analysis, horizontal pleiotropy analysis, and leave-one-out sensitivity analysis.

**Conclusion:**

This study has confirmed a causal relationship between HMGCR and PSC risk, suggesting statins targeting HMGCR could enhance PSC patient outcomes.

## 1 Introduction

Primary sclerosing cholangitis (PSC) is a chronic cholestatic liver disease characterized by injury to the intrahepatic, extrahepatic, or both bile ducts [1]. Although the exact pathogenesis of PSC is unknown, various mechanistic theories have been proposed, involving genetic, immunologic, and environmental factors in the disease’s development [2]. No single drug or treatment has demonstrated a capacity to prolong transplant-free survival in PSC [1]. Despite extensive research on ursodeoxycholic acid (UDCA) as the most commonly used drug, evidence supporting its long-term benefits remains unclear, and its use remains controversial [3,4].

PSC patients are known to have increased lipid levels in serum [5]. Jorgensen et al. demonstrated that hypercholesterolemia is prevalent among patients with PSC, and its occurrence tends to increase with the severity of the disease [6]. Gandelman et al. suggested a disease-specific mechanism for dyslipidemia in PSC based on a case report of a patient with severe dyslipidemia [7]. This case highlights a potential association between the patient’s lipid abnormalities and hepatic impairment in PSC, indicating a disease-specific link. Emerging evidence suggests the potential benefits of statin use in chronic liver diseases [8,9]. A recent meta-analysis indicated that statin use might be linked to a reduced risk of liver failure, death, and portal hypertension [8]. Statins function by inhibiting 3-hydroxy-3-methylglutaryl coenzyme A reductase (HMGCR), thereby reducing serum and bile cholesterol levels [10]. Moreover, statins exhibit anti-inflammatory effects, mitigating oxidative stress and inflammation, enhancing nitric oxide synthesis, and improving endothelial function [11]. Additionally, statins have a positive impact on liver inflammation and fibrosis [12]. Currently, only one relevant retrospective study suggests that statins might improve the prognosis of PSC patients [13]. However, despite 70% of PSC patients having concurrent inflammatory bowel disease (IBD) [14], the inclusion of patients with cholangitis and concomitant IBD as PSC in this study has rendered the results less convincing. In summary, the use of statin drugs for treating PSC has not yet gained widespread recognition.

Proprotein convertase subtilis kexin 9 (PCSK9) is a serine protease that plays a crucial role in regulating low-density lipoprotein cholesterol (LDL-C) metabolism, emerging as a key target for cholesterol-lowering therapies [15]. Furthermore, inhibitors of HMGCR and PCSK9 have been extensively employed in the treatment of coronary heart disease (CHD) [16]. Recent discoveries have highlighted that PCSK9 can stimulate the secretion of inflammatory cytokines by monocyte-macrophages through pathways such as TLR4/NF-κB, contributing to inflammation [17]. Additionally, PCSK9 inhibitors have shown therapeutic effects in the autoimmune disease systemic lupus erythematosus [16]. However, the therapeutic role of PCSK9 inhibitors in PSC, also an autoimmune disease, has yet to be explored.

Drug target Mendelian randomization (MR) analysis employs genetic variation simulating the pharmacological inhibition of pharmacogenetic targets as instrumental variables (IVs). Through regression analysis, it can illuminate the effects of long-term drug use and enhance the causal inference regarding the potential impact of these drug gene targets on PSC [15,18]. In this study, we collected recently published genome-wide association study (GWAS) summary-level statistics and investigated the causal relationship between genetically predicted HMGCR and PCSK9 inhibitors and PSC by conducting drug-targeted MR analysis.

## 2 Materials and methods

### 2.1 Data sources

The LDL-C levels data for this analysis were sourced from the largest-to-date GWAS, which included participants of European ancestry from the UK Biobank (*n* = 440,546) [19]. Replication analysis utilized LDL-C data extracted from the most representative GWAS of subjects from the Global Lipids Genetics Consortium (*n* = 173,082) [20]. The PSC dataset (*n* = 14,890) was obtained from the International PSC Study Group, as reported by Ji et al. which investigated the relationship between PSC quantity and single-nucleotide polymorphisms (SNPs) [21]. In this study, we utilized PSC as the result of the drug target MR analysis, with CHD serving as a positive control dataset. The GWAS summary data for CHD were sourced from the Coronary Artery Disease Genome-wide Replication and Meta-analysis plus The Coronary Artery Disease Genetics Consortium, which included 184,305 samples [22]. The SNPs included in the above dataset were obtained from the MRC IEU OPENGWAS database (https://gwas.mrcieu.ac.uk/datasets/) (Table 1). This study was based on publicly available summary data and required no ethics approval or participant consent.

**Table 1:** Details of the genome-wide association studies and datasets used in our analyses

Items	ID	Population	Data sources	PMID
LDL-C	ieu-b-110	European: 440,546	UK Biobank	32203549
LDL-C	ieu-a-300	European: 173,082	GLGC	24097068
CHD	ieu-a-7	Mixed: 184,305	CARDIoGRAMplusC4D	26343387
PSC	ieu-a-1112	Mixed: 14,890	IPSCSG	27992413

CHD, coronary heart disease; PSC, primary sclerosing cholangitis; GLGC, Global Lipids Genetics Consortium; CARDIoGRAMplusC4D, Coronary ARtery Disease Genome wide Replication and Meta-analysis plus The Coronary Artery Disease Genetics; IPSCSG, International PSC Study Group.

### 2.2 Selection of IVs

In this study, IVs were selected as SNPs located within ±100 kb of HMGCR or PCSK9 loci and associated with LDL-C levels. IVs with a *P-*value of less than 5 × 10^−8^ were chosen to enhance the robustness of IV estimation. To mitigate the impact of strong linkage disequilibrium on the results, a linkage disequilibrium threshold was set (*r* ^2^ < 0.3). A total of 19 significant SNPs of HMGCR and 33 SNPs of PCSK9 were identified (Table S1). Pertinent information for each SNP, including the effect allele, effect size (*β*-value), standard error, and *P*-value, was extracted from the MR results. Subsequently, we assessed the strength of the IVs by evaluating the proportion of variation explained (*R* ^2^) and *F*-statistics. A threshold of *F* < 10 was employed to define a “weak IV,” which would be excluded from the study.

### 2.3 Data analysis

HMGCR and PCSK9 inhibitors are widely employed in treating CHD. Consequently, we utilized GWAS summary data for CHD as a positive control to validate the effectiveness of IVs. Initially, we harmonized the exposure-related drug-targeting IVs with the outcome datasets. We then employed the weighted median and inverse-variance weighted (IVW) methods for analysis, with the IVW method being the most commonly used. A *P*-value of less than 0.05 was considered indicative of a causal relationship between exposure and outcome.

Heterogeneity was assessed using MR Egger and IVW methods. Cochrane’s *Q* value was used to evaluate the heterogeneity of the genetic tools, with *P >* 0.05 suggesting no significant heterogeneity. MR Egger regression equation was applied to assess the horizontal pleiotropy of the genetic tools, and *P >* 0.05 indicated the absence of horizontal pleiotropy. Sensitivity analysis was conducted again after removing outliers through the MR-PRESSO test.

**Figure 1:**
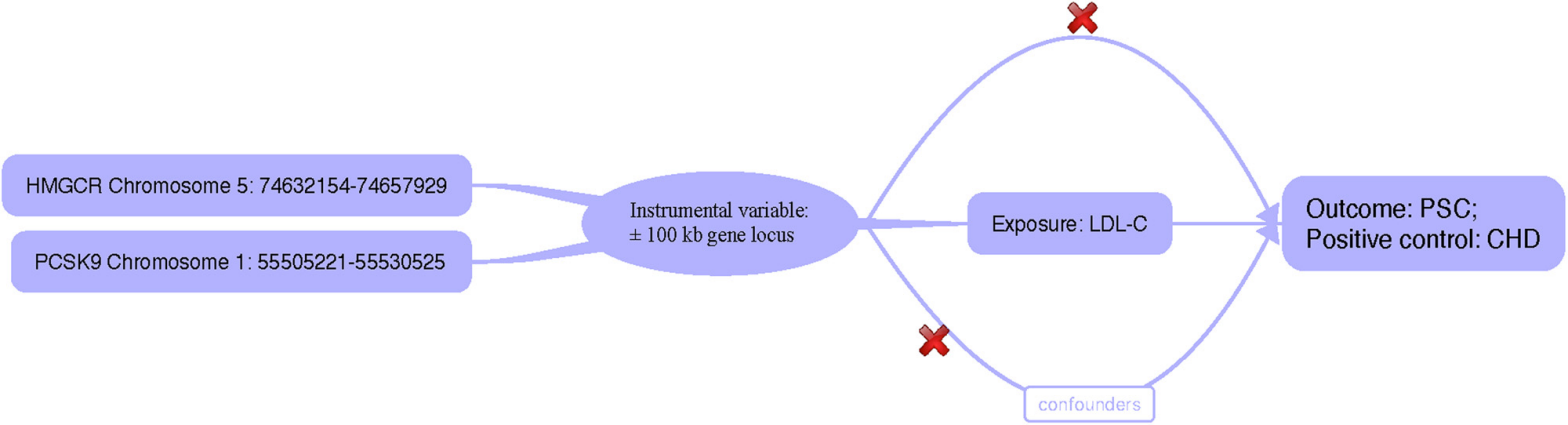
Research overview and design of drug target MR analysis. LDL-C, low-density lipoprotein cholesterol; PCSK9, proprotein convertase subtilisin/kexin type 9; HMGCR, 3-hydroxy-3-methylglutaryl-coenzyme A reductase; CHD, coronary heart disease; PSC, primary sclerosing cholangitis; PCSK9 and HMGCR inhibitors are extensively employed to mitigate the risk of CHD. Consequently, we have designated CHD as a positive control. To substantiate the presence of a causal correlation, it is imperative to adhere to the following conditions: ^†^IVs are unrelated to confounding factors, ^‡^there is a connection between IVs and the exposure factor, and ^§^IVs do not exhibit a direct association with the outcome.


To ensure our results were not significantly influenced by a particular SNP, we utilized the leave-one-out method, sequentially removing each SNP and comparing the results of the IVW method with all variants. The MR hypothesis requires that SNPs are not directly related to the results (Figure 1). Therefore, the online tool PhenoScanner (http://www.phenoscanner.medschl.cam.ac.uk/) was used to identify traits directly related to the tool variable SNP, excluding those related to CHD and PSC. Data analysis was performed on R version 4.2.3 using MRPRESSO and “TwoSampleMR” packages.

**Ethical approval:** This study was based on publicly available summary data and required no ethics approval or participant consent.

## 3 Results

### 3.1 Positive control analysis

**Figure 2:**
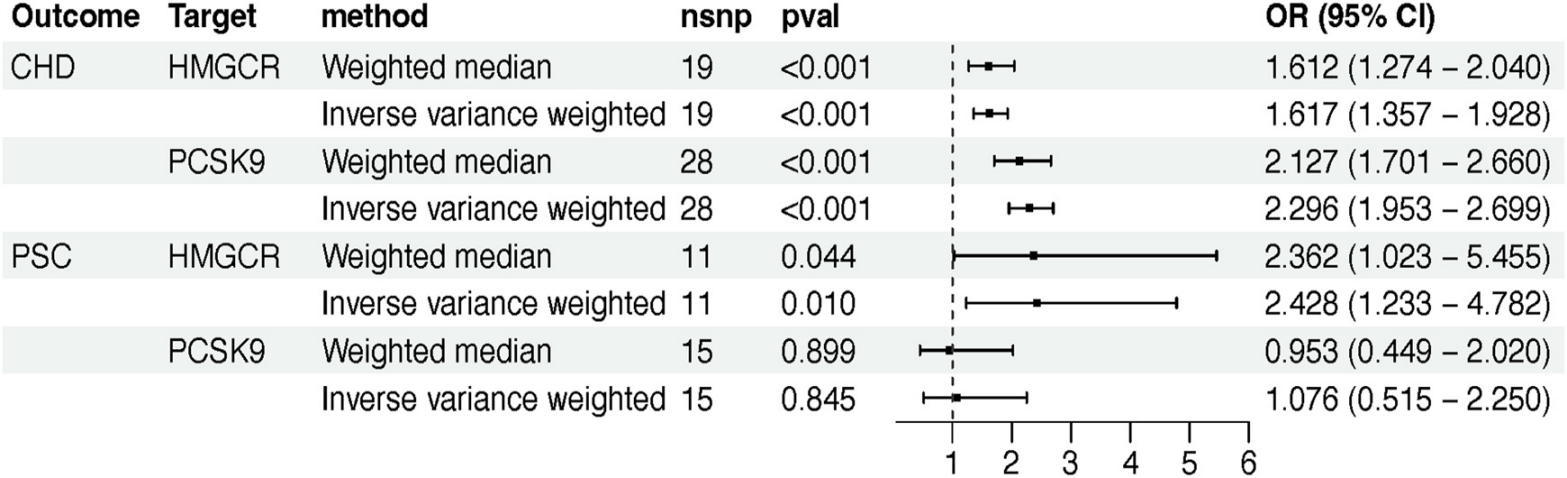
The effect of HMGCR and PCSK9 inhibitor on CHD and PSC. nsnp, number of SNPs; OR, odds ratio; CI, confidence interval; PCSK9, proprotein convertase subtilisin/kexin 9; HMGCR, 3-hydroxy-3-methylglutaryl coenzyme A reductase; CHD, coronary heart disease.

As anticipated, HMGCR significantly increased the risk of CHD in both the IVW method (OR [95%] = 1.62 [1.36–1.93], *P =* 8.29 × 10^−8^) and weighted median method (OR [95%] = 1.61 [1.27–2.04], *P =* 7.09 × 10^−5^) (Figure 2). This effect was similar to that of PCSK9 (IVW: OR [95%] = 2.30 [1.95–2.70], *P =* 7.20 × 10^−24^; weighted median: OR [95%] = 2.13 [1.70–2.66], *P =* 3.54 × 10^−11^) (Figure 2). Upon testing, these results showed no significant heterogeneity or horizontal pleiotropy (*P >* 0.05) (Table S2).

**Figure 3:**
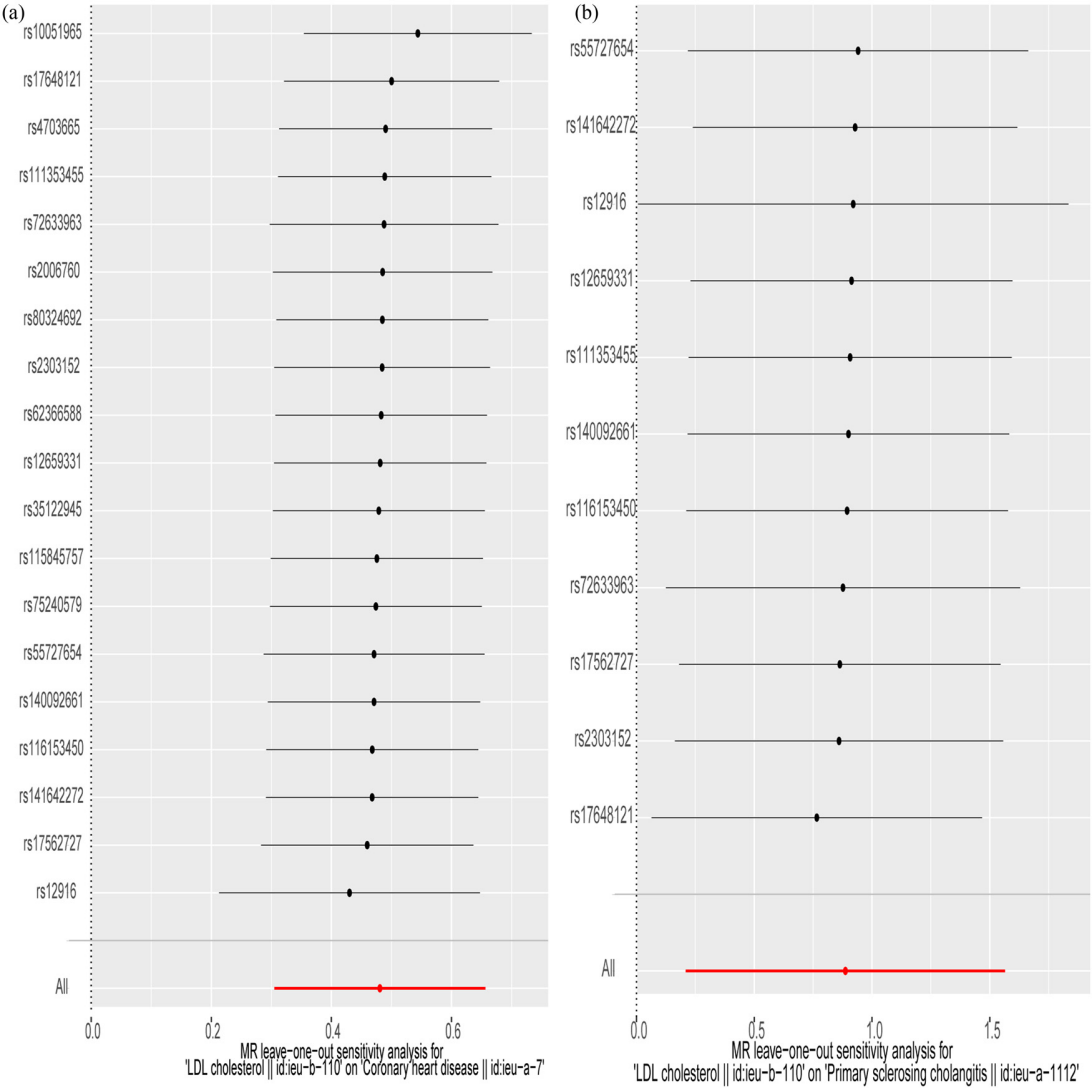
Sensitivity analysis of HMGCR on CHD and PSC. Leave-one-out analysis of HMGCR on CHD (a), PSC (b). The leave-one-out method is used to evaluate the excessive impact of a single SNP on MR analysis if the comprehensive effect of the remaining SNPs is consistent with the main effect after removing one SNP. The IVs select SNPs that are located within ±100 kb of HMGCR loci and related to LDL-C level. SNP, single nucleotide polymorphisms. HMGCR, 3-hydroxy-3-methylglutaryl coenzyme A reductase. CHD, coronary heart disease.

A “leave-one-out” sensitivity analysis was performed to investigate whether the causal associations were driven by any single SNP (Figure 3a). The results indicated that stepwise exclusion of each SNP did not significantly alter the model effect estimates or qualitative inferences. Similar results were obtained by repeating the analysis with another GWAS dataset (Tables S3 and S4), and this result demonstrated no significant heterogeneity or horizontal pleiotropy (Table S5).

### 3.2 The causal relationship between gene‑simulated HMGCR and PCSK9 and PSC

Genetically predicted HMGCR exhibited a pronounced detrimental effect on PSC in both the IVW method (OR [95%] = 2.43 [1.23–4.78], *P =* 0.010) and weighted median method (OR [95%] = 2.36 [1.02–5.45], *P =* 0.04) (Figure 2). However, PCSK9 did not reach statistical significance (IVW: *P =* 0.85; weighted median method: *P =* 0.90) (Figure 2). The robustness of the results was assessed using Cochrane’s *Q* and the MR Egger regression equation, both of which showed no significant heterogeneity or horizontal pleiotropy (*P >* 0.05) (Table S2).

A “leave-one-out” sensitivity analysis was performed to investigate whether the causal associations between HMGCR and PSC were driven by any single SNP. Importantly, both predictive outputs and interpretative conclusions remained robust, irrespective of the omission of any one SNP factor (Figure 3b). Analyses using PhenoScanner indicated that SNPs were primarily associated with blood lipids and body fat content and were not directly related to the outcome variables. Additionally, we used another GWAS dataset for repeated analysis, reaching similar conclusions (Table S4). Furthermore, the results of sensitivity analysis demonstrated no heterogeneity and horizontal pleiotropy in all other outcomes (*P >* 0.05) (Table S5).

## 4 Discussion

Extensive research has explored various treatments for PSC, including UDCA [23], immunosuppressive therapy [24], and antibiotics [25], among others. However, no single intervention has definitively proven to significantly extend transplant-free survival in PSC patients [1]. Notably, statins targeting HMGCR show promise in improving the prognosis of individuals with PSC [13]. However, flaws in the inclusion criteria, specifically categorizing patients with both cholangitis and IBD as having PSC, render the conclusions of this study contentious [26]. In a pioneering approach, our study is the first to use two-sample MR analysis with large-scale GWAS datasets, establishing a causal link between HMGCR and PSC. A series of sensitivity analyses supported the findings mentioned above. In brief, our results suggest that HMGCR inhibition could enhance PSC prognosis, providing robust evidence supporting the therapeutic use of statins in PSC treatment.

PSC is commonly associated with cholestasis and abnormal lipid metabolism [6]. Patients with PSC often exhibit elevated lipid levels. In PSC patients with compensated liver disease, LDL-C levels show a positive correlation with the degree of cholestasis, as indicated by liver biochemistries [5,6]. Additionally, research has demonstrated that UDCA not only alleviates cholestasis symptoms but also has a positive impact on total cholesterol and LDL-C levels in patients with cholestasis [27]. This observation suggests a potential pathogenic pathway in PSC related to lipid metabolism [5].

Statins, commonly employed as lipid-lowering agents, function by inhibiting cholesterol production and reducing serum cholesterol levels through the inhibition of HMGCR [28]. However, their clinical efficacy appears to extend beyond LDL-C reduction, a phenomenon recognized as “statin pleiotropy” [29]. Notably, research has unveiled potent anti-inflammatory properties of statins in the context of Staphylococcus aureus alpha-toxin infection [30]. Additionally, statins have demonstrated the ability to significantly decrease CRP levels in hypercholesterolemic patients over an 8-week period, independent of lipid levels [31]. Two meta-analyses have highlighted the role of statins in attenuating disease activity in rheumatoid arthritis (RA) by lowering serum inflammatory markers and improving symptoms [32,33]. A large population-based nested case-control study has further supported the notion that statin use reduces the risk of RA. Concurrently, evidence suggests that statins may modulate the immune system by regulating T-cell signaling, antigen presentation, immune cell migration, and cytokine production [34]. Their potential therapeutic application extends to various autoimmune diseases, including systemic lupus erythematosus [35], multiple sclerosis [36], and graft-versus-host disease [37]. In our study, we established a causal relationship between HMGCR and PSC through MR analysis, demonstrating that statins, as inhibitors of HMGCR, enhance the prognosis of PSC patients. However, the precise mechanism of action remains unclear. It is plausible that statins exert anti-inflammatory effects by reducing oxidative stress and inflammation, diminishing the activation of inflammatory cells [38,39], and improving endothelial function through increased nitric oxide synthesis, restoration of endothelial cell function, and augmentation of endothelial progenitor cells [40,41]. Additionally, prior research suggests that statins positively impact hepatic inflammation, fibrosis, and hepatic vascular tone. Recent studies have also linked statin use to the risk of fibrosis progression and hepatic decompensation in individuals with chronic liver diseases [8].

It must be acknowledged that our study has certain limitations. First, because MR analysis is merely a method to analyze the causal relationship between exposure and outcome, it cannot replace clinical trials in the real world. Additionally, we failed to elucidate the specific mechanism by which HMGCR inhibitors improve the prognosis of PSC patients. Furthermore, due to genetic heterogeneity among various populations, future research should conduct subgroup analyses in diverse populations to obtain a more comprehensive conclusion.

## 5 Conclusions

This study has established a causal relationship between HMGCR and PSC. The results offer robust evidence supporting the potential enhancement of prognosis in PSC patients through the use of statin drugs targeting HMGCR. It also brings new hope to a large number of PSC patients.

## Supplementary Material

Supplementary Table
