# Supplementary material for: New target-HMGCR inhibitors for the treatment of primary sclerosing cholangitis: A drug Mendelian randomization study
Source: Open Med (Wars). 2024 Jul 19;19(1):20240994. doi: 10.1515/med-2024-0994 (PMC11260000; doi:10.1515/med-2024-0994)
Supplement: Supplementary Table [file med-2024-0994-sm.pdf]

Supplementary material

Table S1: The detail of instrumental variable corresponding to PCSK9 and HMGCR

	SNP	Chr	Pos	Beta	SE	*p*-value	Effect alleles	Other alleles
HMCGR	rs75240579	5	74,624,484	−0.0372115	0.00487202	2.20×10^{-14}	T	C
HMCGR	rs2006760	5	74,562,029	0.03556	0.00261075	3.00×10^{-42}	G	C
HMCGR	rs62366588	5	74,664,987	−0.0271295	0.00433093	3.70×10^{-10}	A	C
HMCGR	rs141642272	5	74,615,209	0.0532822	0.00653971	3.70×10^{-16}	C	G
HMCGR	rs55727654	5	74,651,864	0.042154	0.0029315	6.90×10^{-47}	A	G
HMCGR	rs111353455	5	74,623,949	0.0243909	0.00372718	6.00×10^{-11}	A	G
HMCGR	rs2303152	5	74,641,707	0.0333589	0.00345272	4.40×10^{-22}	A	G
HMCGR	rs116153450	5	74,729,433	−0.0303618	0.00499242	1.20×10^{-9}	A	C
HMCGR	rs12916	5	74,656,539	0.0621175	0.00212705	1.70×10^{-187}	C	T
HMCGR	rs17562727	5	74,682,474	0.0394972	0.00635898	5.30×10^{-10}	C	T
HMCGR	rs80324692	5	74,717,761	−0.0260509	0.00385694	1.40×10^{-11}	T	C
HMCGR	rs115845757	5	74,563,700	0.048608	0.00785612	6.10×10^{-10}	A	G
HMCGR	rs17648121	5	74,650,106	0.0619849	0.00619367	1.40×10^{-23}	T	C
HMCGR	rs140092661	5	74,682,600	0.0329927	0.00582168	1.50×10^{-8}	T	A
HMCGR	rs12659331	5	74,757,657	0.0251785	0.00459392	4.20×10^{-8}	C	A
HMCGR	rs72633963	5	74,630,829	0.0564278	0.00316653	4.90×10^{-71}	A	G
HMCGR	rs10051965	5	74,560,487	0.0410063	0.00216518	5.40×10^{-80}	T	C
HMCGR	rs35122945	5	74,610,293	−0.0281057	0.00423755	3.30×10^{-11}	C	A
HMCGR	rs4703665	5	74,602,898	0.0244938	0.00297742	1.90×10^{-16}	C	T
PCSK9	rs6691964	1	55,433,978	−0.0234719	0.00358388	5.80×10^{-11}	A	G
PCSK9	rs556369867	1	55,491,135	0.0175746	0.00243048	4.80×10^{-13}	T	C
PCSK9	rs72909541	1	55,494,301	−0.0334061	0.00501479	2.70×10^{-11}	T	C
PCSK9	rs150119739	1	55,520,938	0.0452728	0.00520209	3.20×10^{-18}	A	G
PCSK9	rs7525503	1	55,522,558	0.0454642	0.0075822	2.00×10^{-9}	T	G
PCSK9	rs11587071	1	55,522,674	−0.0282322	0.00279415	5.30×10^{-24}	T	C
PCSK9	rs10493176	1	55,538,552	−0.0531381	0.00394676	2.60×10^{-41}	G	T
PCSK9	rs3976734	1	55,489,960	−0.0297494	0.00231882	1.10×10^{-37}	G	A
PCSK9	rs200730299	1	55,491,853	−0.0543492	0.00278155	5.10×10^{-85}	C	A
PCSK9	rs17192725	1	55,496,131	0.0305717	0.00365832	6.40×10^{-17}	A	G
PCSK9	rs17111503	1	55,503,448	0.0406795	0.00235743	1.00×10^{-66}	G	A
PCSK9	rs7546522	1	55,516,713	−0.0168117	0.00295297	1.20×10^{-8}	T	C
PCSK9	rs2483205	1	55,518,316	−0.0295845	0.00214514	2.90×10^{-43}	T	C
PCSK9	rs11583974	1	55,551,718	0.0314531	0.00517068	1.20×10^{-9}	A	G
PCSK9	rs56349475	1	55,576,102	−0.0475957	0.00671909	1.40×10^{-12}	C	T

(Continued)

Table S1: *Continued*

	SNP	Chr	Pos	Beta	SE	*p*-value	Effect alleles	Other alleles
PCSK9	rs79396670	1	55,588,142	−0.0336489	0.00562029	2.10×10^{-9}	A	G
PCSK9	rs146273942	1	55,453,841	−0.0538858	0.00722418	8.70×10^{-14}	A	G
PCSK9	rs2479420	1	55,492,190	−0.0283879	0.0023826	9.90×10^{-33}	T	C
PCSK9	rs11810371	1	55,496,861	−0.0294547	0.00507333	6.40×10^{-9}	A	G
PCSK9	rs11591147	1	55,505,647	−0.348456	0.00793088	1.00×10^{-200}	T	G
PCSK9	rs11206513	1	55,507,649	0.0316517	0.0021463	3.20×10^{-49}	T	C
PCSK9	rs11206517	1	55,526,428	0.0680285	0.00580615	1.00×10^{-31}	G	T
PCSK9	rs2495517	1	55,448,842	0.0177548	0.0025792	5.80×10^{-12}	G	A
PCSK9	rs12732125	1	55,470,153	−0.10344	0.0073736	1.00×10^{-44}	T	C
PCSK9	rs2479395	1	55,484,582	0.0125674	0.00221762	1.50×10^{-8}	C	T
PCSK9	rs77875082	1	55,485,042	0.0481535	0.00605559	1.80×10^{-15}	A	G
PCSK9	rs41294821	1	55,513,183	−0.0386615	0.00705365	4.20×10^{-8}	T	C
PCSK9	rs472495	1	55,521,313	0.0425743	0.00218093	7.30×10^{-85}	T	G
PCSK9	rs530804537	1	55,583,210	−0.192336	0.00997554	7.80×10^{-83}	A	G
PCSK9	rs55637835	1	55,466,303	−0.0187129	0.00324835	8.40×10^{-9}	T	C
PCSK9	rs12739979	1	55,496,648	−0.0202563	0.00254032	1.50×10^{-15}	T	C
PCSK9	rs72660548	1	55,500,978	0.0509816	0.00777535	5.50×10^{-11}	G	C
PCSK9	rs45613943	1	55,518,622	−0.0340702	0.0048672	2.60×10^{-12}	C	T

PCSK9, proprotein convertase subtilisin/kexin 9; HMGCR, 3-hydroxy-3-methylglutaryl coenzyme A reductase.

Table S2: The result of heterogeneity test and horizontal pleiotropic test

Outcomes	Drug Target	Heterogeneity test				Horizontal pleiotropic test		
		Method	*Q*	Q_df	Q_pval	egger_intercept	SE	p-value
CHD	HMGCR	MR Egger	16.345	17	0.499	−0.008	0.013	0.550
		IVW	16.718	18	0.543			
	PCSK9	MR Egger	25.777	26	0.475	0.005	0.006	0.400
		IVW	26.498	27	0.491			
PSC	HMGCR	MR Egger	2.810	9	0.971	−0.034	0.056	0.564
		IVW	3.170	10	0.977			
	PCSK9	MR Egger	20.697	13	0.079	0.015	0.028	0.595
		IVW	21.170	14	0.097			

IVM, inverse-variance weighted; CHD, coronary heart disease; PSC, primary sclerosing cholangitis.

Table S3: The detail of instrumental variable corresponding to HMGCR and PCSK9 for repeated analysis

	SNP	Chr	Pos	Beta	SE	*p*-value	Effect alleles	Other alleles
HMGCR	rs3857388	5	74,620,377	0.0421	0.0059	2.20×10^{-11}	C	T
HMGCR	rs10515198	5	74,641,560	0.0599	0.0061	5.99×10^{-22}	A	G
HMGCR	rs12659791	5	74,757,758	0.0433	0.005	1.42×10^{-18}	C	T
HMGCR	rs72633962	5	74,569,028	0.06	0.0072	3.33×10^{-15}	C	T
HMGCR	rs3804231	5	74,696,779	0.0642	0.0053	1.88×10^{-29}	A	G
HMGCR	rs10066707	5	74,560,579	0.0497	0.0054	2.97×10^{-19}	A	G
HMGCR	rs2006760	5	74,562,029	0.0533	0.0076	1.67×10^{-13}	G	C
HMGCR	rs12916	5	74,656,539	0.0733	0.0038	7.79×10^{-78}	C	T
PCSK9	rs2495495	1	55,496,556	−0.0342	0.0059	3.52×10^{-8}	C	T
PCSK9	rs2495477	1	55,518,467	−0.064	0.0054	7.28×10^{-30}	G	A
PCSK9	rs2479409	1	55,504,650	−0.0642	0.0041	2.51×10^{-50}	A	G
PCSK9	rs12067569	1	55,528,629	0.0885	0.01	1.97×10^{-17}	A	G
PCSK9	rs10493176	1	55,538,552	−0.0776	0.0102	2.54×10^{-14}	G	T
PCSK9	rs11591147	1	55,505,647	−0.497	0.018	8.57×10^{-143}	T	G
PCSK9	rs4927193	1	55,509,872	−0.0352	0.0056	4.27×10^{-11}	C	T
PCSK9	rs11583974	1	55,551,718	0.0646	0.0117	3.95×10^{-9}	A	G
PCSK9	rs2479394	1	55,486,064	−0.0386	0.0041	1.58×10^{-19}	A	G
PCSK9	rs11206510	1	55,496,039	−0.0831	0.005	2.38×10^{-53}	C	T
PCSK9	rs572512	1	55,517,344	0.0478	0.0047	5.31×10^{-26}	T	C
PCSK9	rs11206514	1	55,516,004	0.0507	0.0041	9.95×10^{-33}	A	C
PCSK9	rs585131	1	55,524,116	0.0637	0.005	2.70×10^{-35}	T	C

PCSK9, proprotein convertase subtilisin/kexin 9; HMGCR, 3-hydroxy-3-methylglutaryl coenzyme A reductase.

Table S4: Investigating the Impact of HMGCR and PCSK9 Inhibitors on CHD and PSC through Repeated Analyses

Outcome	Target	Method	nsnp	*p*-value	or	or_lci95	or_uci95
CHD	HMGCR	Weighted median	7	2.61×10^{-5}	1.496	1.240	1.805
		IVW	7	2.18×10^{-6}	1.444	1.240	1.682
	PCSK9	Weighted median	13	6.82×10^{-10}	1.685	1.428	1.989
		IVW	13	6.58×10^{-15}	1.676	1.472	1.908
PSC	HMGCR	Weighted median	6	0.023*	2.092	1.107	3.955
		IVW	6	0.006**	2.195	1.258	3.830
	PCSK9	Weighted median	12	0.758	1.073	0.684	1.684
		IVW	12	0.191	1.292	0.880	1.895

IVM, inverse-variance weighted; CHD, coronary heart disease; PSC, primary sclerosing cholangitis.

*: $P < 0.05$; **: $P < 0.01$.

Table S5: The result of heterogeneity test and horizontal pleiotropic test for repeated analysis

Outcomes	Drug Target	Heterogeneity test				Horizontal pleiotropic test		
		**Method**	***Q***	**Q_df**	**Q_pval**	**egger_intercept**	**SE**	**p-value**
CHD	HMGCR	MR Egger	3.162	5	0.675	−0.029	0.023	0.272
		IVW	4.685	6	0.585			
	PCSK9	MR Egger	16.899	11	0.111	−0.003	0.009	0.768
		IVW	17.040	12	0.148			
PSC	HMGCR	MR Egger	0.474	4	0.976	0.007	0.086	0.943
		IVW	0.480	5	0.993			
	PCSK9	MR Egger	9.431	10	0.492	0.049	0.023	0.054
		IVW	14.200	11	0.222			

IVM, inverse-variance weighted; CHD, coronary heart disease; PSC, primary sclerosing cholangitis.
